# COVID-19 vaccination campaigns in fragile and conflict-affected settings, Somalia

**DOI:** 10.2471/BLT.23.291105

**Published:** 2024-07-11

**Authors:** Muhammad Farid, Abdulrazak Ibrahim, Hamayoun Mohammad, Quamrul Hassan, Mohamed Abdullahi Omar, Mohamed Abdulrahman Ismael, Abdifatah Mohamed Shidane, Mohamed Farah Mohamud, Mukhtar Shube, Mustafe Awil Jama, Patience Musanhu, Rehan Hafiz, Sk Md Mamunur Rahman Malik

**Affiliations:** a World Health Organization, Country Office, Chelsea compound, Aden Adde International Airport Road, Benadir region, Mogadishu, Somalia.; b World Health Organization, Regional Office for the Eastern Mediterranean, Cairo, Egypt.; c Federal Ministry of Health, Mogadishu, Somalia.; d Gavi, the Vaccine Alliance, Geneva, Switzerland.; e Bill & Melinda Gates Foundation, London, England.

## Abstract

**Problem:**

By 31 December 2021, only 5.5% (861 879/15 670 530) of the Somali population had been fully vaccinated against coronavirus disease 2019 (COVID-19).

**Approach:**

To rapidly increase COVID-19 vaccine coverage in 2022, the health ministry and its partners (World Health Organization and United Nations Children’s Fund) adopted a more responsive strategy. This strategy included careful microplanning, better targeting of populations and providing people-centred vaccination services close to their homes. These services were combined with childhood vaccination and basic health-care provision using the existing polio network and community health workers. Additionally, a digital tool for recording COVID-19 vaccination data and a mobile phone-based electronic registration system were introduced.

**Local setting:**

Somalia, a fragile and conflict-affected state, faced challenges when implementing COVID-19 vaccination, including inexperience in managing mass adult vaccination, inadequate infrastructure and health workforce. Furthermore, insecurity in some areas and severe drought resulted in large-scale displacement of people.

**Relevant changes:**

The implementation of a more context-specific strategy helped Somalia reach substantially more people with COVID-19 vaccination and 42.1% coverage by 31 December 2022. Additionally, 84 600 zero-dose children received their first childhood vaccine during the integrated campaigns. The increased coverage has led to public health benefits that outweigh the investment in the COVID-19 vaccination campaigns.

**Lessons learnt:**

Successful roll-out of adult vaccination is achievable even in a fragile and conflict-affected setting through implementation of a tailored contextualized approach. Key factors include good microplanning, use of digital tools, better population-targeting, bundling vaccines together and delivering vaccination services close to people’s homes.

## Résumé

**Problème:**

Au 31 décembre 2021, seulement 5,5% (861 879/15 670 530) des Somaliens étaient totalement vaccinés contre la maladie à coronavirus 2019 (COVID-19).

**Approche:**

Afin d'accroître rapidement la couverture vaccinale contre la COVID-19 en 2022, le Ministère de la Santé et ses partenaires (l'Organisation mondiale de la Santé et le Fonds des Nations Unies pour l'enfance) ont adopté une stratégie plus réactive. Celle-ci comprenait une microplanification minutieuse, un meilleur ciblage des populations et la fourniture de services de vaccination centrés sur les personnes, à proximité de leur domicile. Ces services étaient combinés à une vaccination des enfants et à des soins de base, grâce au réseau actuel de lutte contre la polio et des agents de santé communautaires. En outre, un outil numérique d'acquisition des données relatives à la vaccination contre la COVID-19 ainsi qu'un système d'enregistrement électronique via téléphone mobile ont été déployés.

**Environnement local:**

La Somalie, un État fragile et en conflit, a été confrontée à des difficultés de mise en œuvre de la vaccination contre la COVID-19, notamment en raison de son manque d'expérience dans la gestion de campagnes de vaccination massive des adultes, de ses infrastructures inadaptées et d'une pénurie de soignants. De plus, l'insécurité qui règne dans certaines régions et la sécheresse extrême ont entraîné des déplacements à grande échelle.

**Changements significatifs:**

L'instauration d'une stratégie mieux adaptée au contexte a aidé la Somalie à vacciner nettement plus de gens contre la COVID-19, et à atteindre 42,1% de couverture vaccinale au 31 décembre 2022. Par ailleurs, 84 600 enfants «zéro dose» ont reçu leur premier vaccin pédiatrique durant les campagnes combinées. L'amélioration de la couverture vaccinale a engendré de nombreux bénéfices pour la santé publique, dépassant l'investissement dans les campagnes de vaccination contre la COVID-19.

**Leçons tirées:**

En définissant une approche contextualisée et sur mesure, il est possible de procéder à la vaccination des adultes, même dans une région fragile et en conflit. Parmi les facteurs clés figurent une bonne microplanification, le recours aux outils numériques, un meilleur ciblage des populations, le regroupement des vaccins et la mise à disposition de services de vaccination non loin des habitations.

## Resumen

**Situación:**

A 31 de diciembre de 2021, solo el 5,5% (861 879/15 670 530) de la población somalí había sido completamente vacunada contra la enfermedad por coronavirus de 2019 (COVID-19).

**Enfoque:**

Para aumentar rápidamente la cobertura de la vacuna contra la COVID-19 en 2022, el Ministerio de Sanidad y sus asociados (la Organización Mundial de la Salud y el Fondo de las Naciones Unidas para la Infancia) adoptaron una estrategia más receptiva. Esta estrategia incluía una cuidadosa microplanificación, una mejor selección de las poblaciones y la prestación de servicios de vacunación centrados en las personas y cercanos a sus hogares. Los servicios se combinaron con la vacunación infantil y la prestación de atención sanitaria básica mediante la red antipoliomielítica existente y los agentes de salud comunitarios. Además, se introdujeron una herramienta digital para registrar los datos de vacunación contra la COVID-19 y un sistema de registro electrónico por teléfono móvil.

**Marco regional:**

Somalia, un estado frágil y afectado por conflictos, atravesó dificultades a la hora de implementar la vacunación contra la COVID-19, como la inexperiencia en la gestión de la vacunación masiva de adultos, la falta de infraestructuras y de personal sanitario. Asimismo, la inseguridad en algunas regiones y la grave sequía provocaron desplazamientos de población a gran escala.

**Cambios importantes:**

La implementación de una estrategia más adaptada al contexto ayudó a Somalia a llegar a un número mucho mayor de personas con la vacunación contra la COVID-19 y a una cobertura del 42,1% a 31 de diciembre de 2022. Además, 84 600 niños con dosis cero recibieron su primera vacuna infantil durante las campañas integradas. El aumento de la cobertura ha propiciado beneficios para la salud pública que superan la inversión en las campañas de vacunación contra la COVID-19.

**Lecciones aprendidas:**

El despliegue exitoso de la vacunación de adultos es posible incluso en un entorno frágil y afectado por conflictos mediante la implementación de un enfoque contextualizado y adaptado. Los factores clave consisten en una buena microplanificación, el uso de herramientas digitales, una mejor orientación de la población, la agrupación de vacunas y la prestación de servicios de vacunación cerca de los hogares.

## ملخص

**المشكلة:**

بحلول يوم 31 ديسمبر/كانون أول عام 2021، تم تطعيم %5.5 فقط (861879/15670530) من السكان في الصومال بشكل كامل ضد مرض فيروس كورونا 2019 (كوفيد 19).

**الأسلوب:**

من أجل زيادة تغطية لقاح كوفيد 19 بسرعة في عام 2022، اعتمدت وزارة الصحة وشركاؤها (منظمة الصحة العالمية وصندوق منظمة الأمم المتحدة للطفولة) استراتيجية أكثر استجابة. وشملت هذه الاستراتيجية التخطيط الجزئي المحترز، واستهداف السكان بشكل أفضل، وتقديم خدمات التطعيم التي تركز على السكان بالقرب من منازلهم. وتم جمع هذه الخدمات مع تطعيم الأطفال وتقديم الرعاية الصحية الأساسية مع الاستعانة بالشبكة الحالية لشلل الأطفال، والعاملين في مجال الصحة المجتمعية. بالإضافة إلى ذلك، تم طرح أداة رقمية لتسجيل بيانات التطعيم ضد كوفيد 19، ونظام للتسجيل إلكتروني قائم على الهاتف المحمول.

**المواقع المحلية:**

الصومال هي دولة هشة متأثرة بالصراع، واجهت تحديات عند تنفيذ التطعيم ضد كوفيد 19، وشملت هذه التحديات قلة الخبرة في إدارة التطعيم الجماعي للبالغين، وعدم ملاءمة البنية التحتية والقوى العاملة في القطاع الصحي. وعلاوة على ذلك، أدى انعدام الأمن في بعض المناطق والجفاف الشديد إلى تهجير ضخم للسكان.

**التغيّرات ذات الصلة:**

إن تنفيذ استراتيجية أكثر ملاءمة للوضع في الصومال، ساعد على وصول التطعيم ضد فيروس كورونا إلى عدد أكبر بكثير من السكان، وتغطية بنسبة %42.1 بحلول 31 ديسمبر/كانون ثاني عام 2022. بالإضافة إلى ذلك، حصل 84600 طفل على جرعة صفرية من أول لقاح للأطفال خلال الحملات المتكاملة. وقد أدت زيادة التغطية إلى فوائد للصحة العامة تفوق الاستثمار في حملات التطعيم ضد كوفيد 19.

**الدروس المستفادة:**

يمكن تحقيق التنفيذ الناجح لتطعيم البالغين حتى في البيئات الهشة والمتأثرة بالصراعات من خلال تنفيذ أسلوب مخصص حسب الأوضاع. وتشمل العوامل الرئيسية التخطيط الجزئي الجيد، واستخدام الأدوات الرقمية، والاستهداف الأفضل للسكان، وتجميع اللقاحات معًا، وتقديم خدمات التطعيم بالقرب من منازل السكان.

## 摘要

**问题:**

截至 2021 年 12 月 31 日，仅 5.5% (861,879/15,670,530) 的索马里民众完全接种了新型冠状病毒肺炎 (COVID-19) 疫苗。

**方法:**

为了在 2022 年迅速提高 COVID-19 疫苗的接种率，卫生部及其合作伙伴（世界卫生组织和联合国儿童基金会）采取了一项响应率更高的战略。这一战略包括精心制定微观计划、更好地定位目标人群以及在居民住所附近提供以人为本的疫苗接种服务。通过利用现有的脊髓灰质炎网络和社区卫生工作者，将这些服务与儿童疫苗接种和基本卫生保健服务结合起来。此外，还引入了用于记录 COVID-19 疫苗接种数据的数字工具和基于手机的电子化登记系统。

**当地状况:**

索马里是一个脆弱且受冲突影响的国家，在实施 COVID-19 疫苗接种方面面临着诸多挑战，包括缺乏管理大规模成人疫苗接种的经验、基础设施和卫生人力资源不足。此外，一些地区的不安全状况和严重干旱导致大批民众流离失所。

**相关变化:**

实施更具针对性的战略帮助索马里实现了大幅提高 COVID-19 疫苗接种率的目标，截至 2022 年 12 月 31 日，该疫苗接种率达到了 42.1%。此外，在综合性疫苗接种活动中，有 84,600 名零剂次儿童接种了第一剂儿童疫苗。接种率提高所带来的公共卫生效益远远大于在 COVID-19 疫苗接种活动方面的投资。

**经验教训:**

即使是在脆弱和受冲突影响的环境下，通过实施有针对性、因地制宜的方法，也可以成功开展成人疫苗接种活动。关键因素包括精心制定微观计划、使用数字工具、更好地定位目标人群、将疫苗进行捆绑接种以及在居民住所附近提供疫苗接种服务。

## Резюме

**Проблема:**

К 31 декабря 2021 года только 5,5% (861 879/15 670 530) населения Сомали были полностью вакцинированы против коронавирусной инфекции 2019 года (COVID-19).

**Подход:**

Для быстрого увеличения охвата вакциной COVID-19 в 2022 году Министерство здравоохранения и его партнеры (Всемирная организация здравоохранения и Детский фонд Организации Объединенных Наций) разработали более оперативную стратегию. Эта стратегия включала тщательное микропланирование, более точное определение целевых групп населения и предоставление услуг по вакцинации, ориентированных на людей, вблизи мест их проживания. Эти услуги сочетались с вакцинацией детей и оказанием базовой медицинской помощи с использованием существующей сети по борьбе с полиомиелитом и медицинских работников на уровне общин. Кроме того, был внедрен цифровой инструмент для регистрации данных о вакцинации COVID-19 и система электронной регистрации с помощью мобильного телефона.

**Местные условия:**

Сомали, нестабильное и пострадавшее от конфликта государство, столкнулось с проблемами при проведении вакцинации COVID-19, включая неопытность в организации массовой вакцинации взрослого населения, неадекватную инфраструктуру и нехватку медицинских кадров. Кроме того, отсутствие безопасности в некоторых районах и сильная засуха привели к массовому вынужденному переселению людей.

**Осуществленные перемены:**

Реализация стратегии, в большей степени учитывающей конкретные условия, помогла Сомали охватить вакцинацией COVID-19 значительно больше людей, и к 31 декабря 2022 года охват достиг 42,1%. Кроме того, в ходе объединенных кампаний первую детскую вакцину получили 84 600 детей, ранее не вакцинированных. Увеличение охвата привело к положительным результатам в области общественного здравоохранения, которые превосходят инвестиции в кампании по вакцинации COVID-19.

**Выводы:**

Успешное внедрение вакцинации взрослых возможно даже в нестабильной и затронутой конфликтом обстановке благодаря применению индивидуального подхода с учетом конкретных условий. Ключевыми факторами являются: правильное микропланирование, использование цифровых инструментов, более точная адресация населения, объединение вакцин в пакеты и предоставление услуг вакцинации в непосредственной близости от мест проживания людей.

## Introduction

Somalia received its first 300 000 doses of coronavirus disease 2019 (COVID-19) vaccines from the COVID-19 Vaccines Global Access (COVAX) initiative on 15 March 2021,^1^ and the first vaccination campaign started on 16 March 2021. The Somali government, which had never organized adult vaccination before, conducted five COVID-19 vaccine campaigns in 2021, using a two-dose regimen and prioritizing elderly people, health workers and people with chronic conditions. The government deployed 400 mobile vaccination teams, each with two locally recruited vaccinators, two data entry personnel and one social mobilizer. Around 400 fixed primary health-care centres and hospitals also provided COVID-19 vaccination. However, by 31 December 2021, only 5.5% (861 879/15 670 530) of the population were fully vaccinated,^2^ which is lower than the World Health Organization (WHO) global goal of 40% total population coverage. 

The campaign faced numerous challenges and reasons for the low coverage. First, due to insufficient funds and unpredictable vaccine supply, the community outreach and mobile vaccination posts were not set up in sufficient numbers, leaving services unavailable near communities. Second, 38.2% (564/1476) of health workers reported COVID-19 vaccine hesitancy.^3^ Third, the prioritized populations only represented an estimated 19.6% of the total population. Fourth, the campaigns lacked detailed microplanning and digital real-time monitoring of uptake. Fifth, fragile federal structure and poor communication between federal and state ministries, as well as between line ministries led to subpar coordination. Sixth, the vaccination services were only available between morning and noon, preventing working people from attending. Finally, conflict and climatic shocks, which lead to many internally displaced people, aggravated the challenges.^4^

Here we describe how the health ministry and its partners (WHO and United Nations Children’s Fund) devised a more responsive strategy to accelerate COVID-19 vaccination and reach 40% population coverage by 31 December 2022.

## Local setting

Somalia, with a population of about 15.6 million, is a fragile and conflict-affected country.^5^ The prolonged humanitarian crisis has resulted in a weakened, fragmented and under-funded health system. The country has among the lowest universal health services coverage (UHC) indexes (27 out of 100)^6^ and health workforce densities (one doctor, nurse or midwife per 1000) in the world.^3^

Additionally, 25.6% (4 011 655/15 670 530) of the population is nomadic^7^ and large areas of the country face security threats from militias. When COVID-19 vaccination started, about 3.8 million people were internally displaced due to conflict, violence and drought.^8^^,^^9^ During 2021 and 2022, Somalia experienced severe drought, leading to the mass displacement of people seeking food aid; 42.8% (6 700 000/15 670 530) of the population faced acute food insecurity.^8^

During the COVID-19 pandemic, essential childhood vaccination services were disrupted, there were outbreaks of measles and^10^^–^^12^ cholera, and vaccine-derived polio virus was circulating.^13^

## Approach

Drawing lessons from the 2021 campaigns and gathering information on what went wrong, the health ministry and its partners planned and implemented a new strategy tailored to the local context, health system capacity and epidemiological situation. This strategy included organizing community outreach services for accelerated vaccination, including eight rounds of mass campaigns, to ensure the country could administer at least 18 750 COVID-19 vaccine doses daily in 2022 (4.8 million doses total) to reach the WHO goal of 40% total population coverage. To achieve this, assuming each team vaccinated 100 people per day, 5 days per week, we locally recruited 1678 teams of 39 623 surge vaccinators to the campaigns. The surge vaccinators were, for example, unemployed graduate nurses from local nursing or midwifery institutes. District-level managers of the expanded programme on immunization provided a one-day training for these surge vaccinators on how to (i) administer intramuscular COVID-19 vaccines from multidose vials without wastage; (ii) administer single-dose vaccines; (iii) administer temperature-sensitive messenger ribonucleic acid (mRNA) vaccines; (iv) understand injection safety; (v) maintain safe waste disposal and vaccine storage; (vi) monitor and report adverse events; (vii) educate the vaccine recipients on identifying adverse events; (viii) enter data for recording and reporting; and (ix) administer childhood vaccines. For the training, surge vaccinators received 15 United States dollars (US$) and were reimbursed for travel costs. During field deployment, they were paid US$ 15 per day plus a lump sum for travel costs.

We estimated operational costs based on past practices. The estimated cost per dose administered was US$ 5, including the cost of delivery, social mobilization and vaccinators. Gavi, the Vaccine Alliance, and other international partners provided funds.

The strategy placed more emphasis on microplanning, a tool used for mass vaccination campaigns. We developed microplans for each community detailing: (i) logistics requirements; (ii) areas with low vaccination coverage and large nomadic and other vulnerable populations; (iii) estimated number of people to be vaccinated in those areas, and funds and human resources required; (iv) delivery schedules; (v) daily activities for each team member; (vi) strategy to involve the local community; (vii) monitoring of targets; and (viii) supervisory activities. To tackle vaccine hesitancy, we developed a health communication strategy to reach local communities by engaging influencers (for example, elderly people, religious leaders and schoolteachers).

Given the reported backslide in routine vaccination from disruption of essential health and vaccination services during the COVID-19 outbreak, we decided to combine the COVID-19 vaccination campaigns with routine childhood vaccination to maximize the investment. We established a mix of 400 fixed vaccination posts and 1678 mobile teams to deliver integrated vaccination services close to where the targeted populations lived (camps for internally displaced people, and markets or other accessible public places for others).

The community was informed through social mobilization and community engagement that vaccination teams were providing COVID-19 vaccines with routine immunization; adults came for COVID-19 vaccines and children from the same family received routine immunization.

A total of 2030 community health workers (CHWs), who support the delivery of basic health services, and WHO’s frontline village polio volunteers, used during mass polio campaigns, were also assigned to make house-to-house visits. Their task was to identify people needing COVID-19 vaccination and children who had never received scheduled childhood vaccines, and refer them to the nearest health centres outreach post or mobile vaccination team. These CHWs and polio volunteers are from the communities they serve. They receive basic training and are paid monthly by their service providers (US$ 130–150).

Given the security situation in Somalia, the inaccessibility of some areas and moving populations, we decided to use single-dose COVID-19 vaccines, allowing people to complete the primary vaccination with one visit. Additionally, vaccine supplies became more predictable in 2022 through advocacy with donors. We distributed necessary supplies (COVID-19 vaccines, cold-chain equipment and safety boxes), and those needed in remote locations were transported by air for speed and security. To rapidly increase vaccination coverage among the general population and address large-scale immunization gaps among other population groups, eligibility to receive COVID-19 vaccination was shifted from only high-risk groups targeted in 2021 to everyone older than 18 years (an estimated 47.1% of the total population) as the country has predominantly a young population with a median age of 15.3 years. However, we prioritized internally displaced people, nomadic and female populations as coverage was low in these groups, as well as their lack of access to routine health care, increased mobility and living in crowded conditions.

To allow real-time monitoring of vaccination uptake, WHO supported the development of a mobile phone-based application with an internet-based interface (Demagi Inc., Cambridge, United States of America) to record and report COVID-19 vaccination data. WHO also supported the government to introduce a mobile telephone-based electronic registration system (CommCare, Demagi Inc.). This system, used by frontline health workers, registered everyone receiving COVID-19 vaccines and allowed local authorities to monitor uptake against targets and quickly deploy additional resources to match demand.

## Relevant changes

**Fig. 1:**
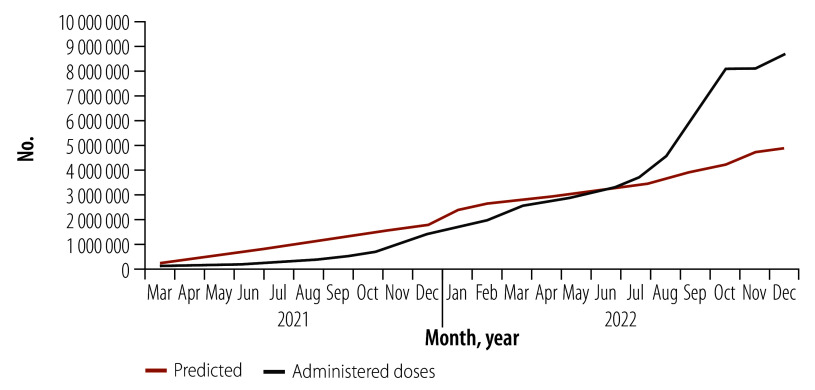
Predicted and actual COVID-19 vaccine doses administered, Somalia 2021–2022

**Fig. 2:**
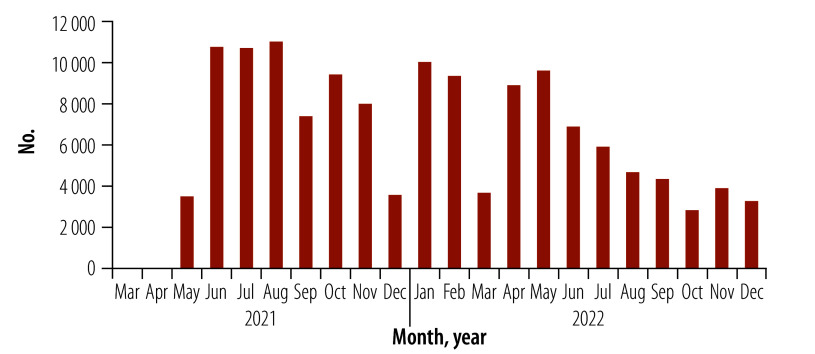
Identified zero-dose children, Somalia 2021–2022

By 31 December 2022, 42.1% (6 597 293/15 670 530) of the population had completed the primary COVID-19 vaccination – 36.6% in 2022 versus 5.5% in 2021 (Table 1). Of those, 1 824 000 were internally displaced people (48.0% of the 3 798 900 displaced population); 641 864 belonged to nomadic populations (16.0% of the 4 011 655 nomadic population); and 6241 were refugees (16.3% of the 38 269 refugee population). By using female vaccinators and CHWs, many women were vaccinated against COVID-19, increasing from  4.0% (235 240/5 831 000) in January 2021 to 46.2% (2 692 260/5 831 000) by December 2022. In 2022, at least 18 750 COVID-19 vaccine doses were administered daily compared with 6688 in 2021. Since COVID-19 vaccination began, 8 676 030 COVID-19 vaccine doses have been administered – 7 010 170 (80.8%) in 2022 (Fig. 1). By integrating routine childhood vaccination and basic health care within the COVID-19 campaigns, 139 350 zero-dose children were identified, with 84 600 (60.7%) receiving their first childhood vaccine in 2022 (Fig. 2). More than 143 600 children received the measles vaccine and 109 000 the third dose of the pentavalent vaccine. About 32 000 women of reproductive age received micronutrient supplements and tetanus vaccination.

**Table 1:** Accelerated COVID-19 vaccination campaigns and outcomes, Somalia, 2021–2022

Year	Doses received, no.	Doses administered, no. (% of doses received)	Accelerated campaigns, no.	Doses administered in all campaigns, median (IQR)	Operational cost per dose administered, US$	Vaccination teams deployed, no.	Additional vaccinators deployed, no.	Eligible population fully vaccinated, (%) (*n* = 15 670 530)
2021	2 484 620	1 665 860 (67.0)	5	122 727 (43 391–166 188)	5	400	2 178	861 879 (5.5)
2022	7 887 026	7 010 170 (88.9)	8	391 386 (216 366–719 652)	3	1 678	39 623	5 735 414 (36.6)
**Total**	**10 371 646^a^**	**8 676 030 (83.6)**	**13**	**NA**	**NA**	**2 078**	**41 801**	**6 597 293 (42.1)**

COVID-19: coronavirus disease 2019; IQR: interquartile range; NA: not applicable; US$: United States dollars.

^a^ The Somali federal health ministry received 5 320 906 doses directly from the COVAX facility as part of COVAX Advance Market Commitment. The remaining 5 050 740 doses of COVID-19 vaccines were received as bilateral donations from France (2 865 600 doses), United States of America (888 780 doses), Sweden (520 800 doses), Türkiye (231 600 doses), China (231 000 doses), Germany (163 200 doses) and United Kingdom of Great Britain and Northern Ireland (149 760 doses).

COVID-19: coronavirus disease 2019

Note: the predicted number shows required doses to be administered to reach the target of > 40% COVID-19 vaccine coverage by 31 December 2022.

## Lessons learnt

The substantial increase in COVID-19 vaccine coverage in Somalia during 2022 suggests that the revised strategy overcame the 2021 barriers. Several factors contributed to this success: (i) better microplanning by vaccination teams at the local level and change in eligibility to receive vaccines; (ii) bringing vaccination services close to where people live; (iii) using a single-dose COVID-19 vaccine; (iv) using mobile applications for real-time registration, targeting, tracking and monitoring; (v) engaging female CHWs and WHO’s polio workforce who linked communities to the health system and increased acceptance of vaccination; and (vi) integrating childhood immunization and basic health-care delivery in the campaigns. The campaigns showed that zero-dose children living in marginalized settings can be reached when vaccination services and delivery of basic health care are provided near where these children live.

The campaigns had several challenges. Before predictable supplies of one-dose vaccines and sufficient funds for operational costs were ensured, accessing the right type and quantity of COVID-19 vaccines was a challenge. However, high-level engagement and advocacy with COVAX helped secure resources. We addressed the security situation and vaccine hesitancy by involving local female CHWs and vaccinators. Their trust within the community helped us reach communities and engage women, improving childhood immunization and vaccination rates among women. Bundling health care with vaccination services and engaging community leaders allowed vaccinators to access security-compromised areas and deliver vaccination services. In settings where the movement of people is an issue, delivering vaccination services near where they live, engaging CHWs to identify people missing vaccines, and combining vaccination with other health-care services can improve coverage (Box 1). Furthermore, the strategy to deliver integrated care using CHWs and involving influencers in decision-making can transform the community-based health-care delivery system in Somalia to improve access to health care including vaccination of people living in hard-to-reach areas. This finding is an important lesson for a country with a low UHC service index and a chronic shortage of health workers to deliver care.

Box 1 **Summary of main lessons learnt**

- Detailed local microplanning and delivering vaccination services close to people’s homes can increase access to vaccines for marginalized populations in hard-to-reach areas and substantially improve vaccination coverage.
- The infrastructure built, tools developed and knowledge gained during the COVID-19 vaccination campaigns can be used to improve routine vaccination delivery systems.
- Engaging local elders to build trust and hiring local female health workers to advocate for health access in security-compromised areas ensured that vaccinators and health workers could reach these areas and overcome service delivery barriers.

COVID-19: coronavirus disease 2019.

Investment in COVID-19 vaccination, particularly in 2022, helped strengthen the delivery of vaccination services in general. The support allowed the Somali government to expand the cold-chain system and build additional storage infrastructure for vaccine delivery to remote settings, especially using solar-powered vaccine carriers and refrigerators. This improvement even allowed the government to roll out heat-sensitive mRNA-based vaccines. These systems are now being used in routine immunization. The investment also allowed the establishment of the first national drug regulatory authority to license vaccines and newly imported drugs. Furthermore, health-care waste management systems and injection safety were improved, and effective pharmacovigilance was instituted to monitor adverse events. These structures are being sustained to support routine immunization, and have improved the delivery of other vaccines used in mass reactive campaigns against outbreaks of measles, cholera and polio. For example, more than 2.9 million and 3.2 million children younger than 5 years were vaccinated against measles and polio, respectively, in 2022.^13^ The recruited surge vaccinators can be used in the childhood vaccination programme when needed to improve childhood vaccination coverage.

The digitalization of the vaccine registration and information systems for tracking vaccines and monitoring vaccination side-effects has been retained for routine immunization. Indeed, an electronic immunization registry and digitized microplanning process is being piloted in Benadir, the main urban city in the country. In the absence of population census data in the country, these electronic tools can identify the number of children eligible for on-time vaccinations, monitor targets and identify zero-dose children and those missing out on one or more vaccines, apart from improving data quality and achieving efficiency.

A case study has shown that the increased COVID-19 vaccination coverage has potentially brought economic benefits for Somalia of up to US$ 316 million^14^ – far more than the vaccine campaign cost which was US$ 21 030 510 for the 2022 campaign. 

Somalia’s experience with COVID-19 vaccination shows that mass vaccination for adults can be successfully implemented even in insecure and hard-to-reach settings, and where other emergencies are ongoing. This experience can inform future pandemic response and control.
